# Supplementary material for: Impact of Early Hemoglobin Levels on Neurodevelopment Outcomes of Two-Year-Olds in Very Preterm Children
Source: Children (Basel). 2023 Jan 25;10(2):209. doi: 10.3390/children10020209 (PMC9955539; doi:10.3390/children10020209)
Supplement: Supplementary file 1 [file children-10-00209-s001.zip › children-2170742-supplementary.pdf]

Table S1 Suppl. Comparison of maternal, obstetrical and neonatal characteristics according to follow-up or non-follow-up at 2 years

	**2-year follow-up** (N = 1490)	**No follow-up** (N = 433)	**p- value**
**Maternal characteristics**			
Age at childbirth, years (mean ± SD)	29.7 (±5.77)	28.3 (±6.53)	< 0.001
Parity (mean ± SD)	1.47 (±1.78)	1.97 (±2.11)	< 0.001
Socio-economic status			$< 10^{-3}$
Higher and intermediate professions	1007 (70.4)	205 (52.5)	
Others	387 (26.8)	160 (40)	
No occupation	41 (2.8)	29 (7.5)	
**Cause of prematurity**			
Primary Causes			0.007
Idiopathic premature labor	561 (38)	161 (38)	
Premature rupture of membranes	326 (22.4)	119 (28.3)	
Vascular pathologies of the placenta			
Without IUGR	222 (15.8)	50 (12.3)	
With IUGR	218 (15.5)	46 (11.4)	
Isolated IUGR	75 (5.4)	27 (6.7)	
Retro-placental hematoma isolated	41 (3)	14 (3.4)	
Metrorrhagia	69 (4.7)	19 (4.5)	0.909
Maternal infections	320 (25.4)	115 (31.8)	0.008
Other causes of premature birth *	105 (7.2)	20 (4.9)	0.065
**Childbirth**			
Weeks GA (mean ± SD)	28.95 (±1.87)	29.01 (±1.84)	0.531
<= 27 Week GA	392 (22.9)	107 (21.4)	
[28-32 [Week GA	1098 (77.1)	326 (78.5)	
**Newborn**			
Birth weight, g (mean ± SD)	1229 (±349)	1254 (±358)	0.146
<= 1000 g	473 (29)	130 (27.2)	
> 1000 g	1017 (71)	303 (72.8)	
Male Gender	774 (52)	218 (50.3)	0.462
Average Apgar at 10 min (average ± SD)	9.19 (±1.40)	9.25 (±1.34)	0.317
**Anemia**			
Hb at birth, g/dL (mean ± SD)	15.47 (±2.31)	15.51 (±2.39)	0.681
Lowest Hb level during hospitalization, g/L (mean ± SD)	10.25 (±2.28)	10.69 (±5.98)	0.011
Hb at discharge g/ L (mean ± SD)	10.99 (±1.97)	11.02 (±1.93)	0.762
Transfusion	616 (39.6)	175 (38.9)	0.767
Number of RBC transfusions (mean ± SD)	2.18 (±1.67)	1.94 (±1.51)	0.058
Erythropoietin	730 (49.6)	205 (51.5)	0.440
**Neonatal morbidity**	213 (14.1)	59 (13.9)	0.908

Data are presented in n (%), unless otherwise indicated. The percentages and p-values are weighted according to GA.
Abbreviations : IUGR: intrauterine growth retardation; SD: standard deviations; Weeks GA: weeks of amenorrhea; Hb: haemoglobin : Hb; RBC: red blood cells.
* Other causes of prematurity including isolated and sporadic explications (acute hepatic steatosis of pregnancy, severe fetal anemia, sickle cell anemia, psychiatric reasons, etc.).
** Neonatal morbidity was defined by: severe bronchopulmonary dysplasia (BDP), severe brain abnormalities (severe periventricular cystic leukomalacia or severe intraventricular haemorrhage (IVH) grade III or IV), necrotizing enteritis colitis (NEC) stage 2 -3 or severe retinopathy of prematurity (ROP)> stage 3.

Figure S1 Suppl. Hb threshold discriminating the outcome at 2 years - ROC curve

Outcome at 2-years: survival without sequelae (without pathological ASQ or cerebral palsy) according to the hemoglobin level at birth : threshold à 15.2 g/dL (AUC > 0.5)

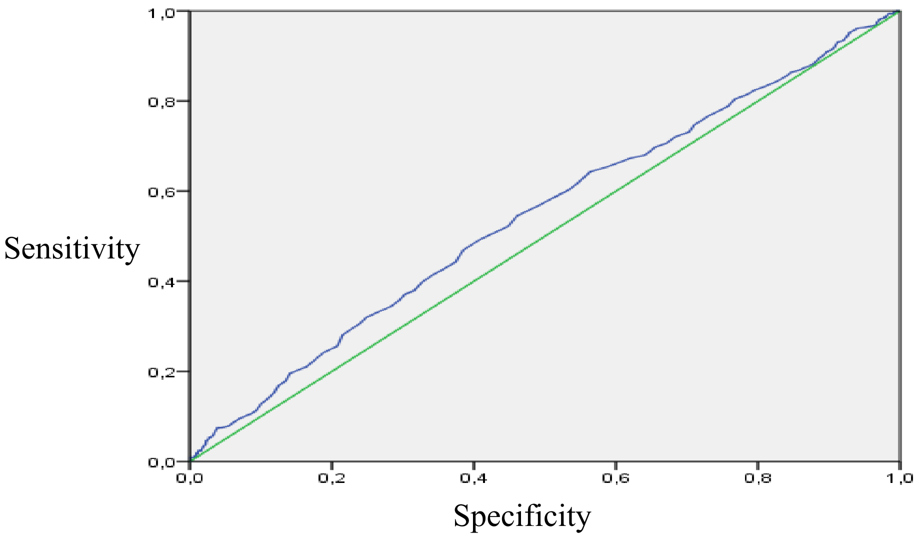

Table S2 Suppl. Curve according to the Hb level at birth

	**Patients** N/total	**AUC** (IC 95%)	**p-value**
**Perinatal prognosis**			
Neonatal morbidity	397/2001	0.605 (0.575-0.636)	$< 10^{-3}$
Death during hospitalization	222/2158	0.632 (0.593-0.671)	$< 10^{-3}$
Neonatal survival without sequelae	501/2057	0.614 (0.586-0.642)	$< 10^{-3}$
**2-year Prognosis**			
Cerebral palsy	80/1588	0.571 (0.512-0.631)	0.031
ASQ <-2DS	835/1512	0.514 (0.485-0.543)	0.354
Living without sequelae (neither ASQ nor PC)	1083/1725	0.540 (0.513-0.568)	0.005

AUC: area under the curve; 95%; CI: 95% confidence interval; ASQ: Ages Stage Questionnaire; CP: cerebral palsy. AUC values are in a range of 0 to 1.A model whose 100% predictions are erroneous has an AUC error of 0.0. If all predictions are correct, the AUC is 1.0. AUC has the following advantages: it is an invariant scale and measures the quality of ranked positions rather than their absolute values. AUC is independent of classification thresholds. It measures the quality of the precision of the model regardless of the classification threshold selected.

Table S3 Suppl. Comparison of maternal, obstetrical and neonatal characteristics according to Hb threshold at birth of 15.2 g/dL.

	**Hb >= 15.2 g/dL** (N = 1144)	**Hb <15.2 g/dL** (N = 1014)	**p-value**
**Maternal characteristics**			
Age at childbirth in years (mean ± SD)	29.45 (±5.95)	29.26 (±6.07)	0.430
Parity (mean ± SD)	1.53 (±1.80)	1.64 (±1.94)	0.095
Tobacco during pregnancy	294 (26.6)	231 (23.9)	0.113
Antidiabetic treatment	47 (4.4)	31 (3.3)	0.164
**Cause of prematurity**			
Primary causes			<0.001
Idiopathic premature labor	358 (31.8)	468 (46.9)	
Premature rupture of membranes	242 (21.8)	254 (25.6)	
Vascular pathologies of the placenta			
Without IUGR	199 (18.2)	86 (9.2)	
With IUGR	202 (18.6)	98 (10.4)	
Isolated IUGR	70 (6.5)	43 (4.6)	
Isolated Retro-placental hematoma	33 (3.1)	31 (3.3)	
Metrorrhagia	44 (3.8)	53 (5.4)	0.038
Maternal infections	209 (21.3)	285 (34.3)	<0.001
Other causes of premature birth *	90 (8.0)	48 (4.9)	0.001
**Childbirth**			
Gestational age, weeks GA (mean ± SD)	29.20 (±1.78)	28.18 (±2.10)	<0.001
<= 27 Week GA	242 (18.4)	436 (38.1)	
[28-32 [Week GA	902 (81.6)	578 (61.9)	
Cesarean	761 (68.1)	594 (60.6)	<0.001
Antenatal corticosteroid	944 (84.2)	769 (77.7)	<0.001
Antenatal magnesium sulfate	128 (11.4)	67 (6.8)	<0.001
Delayed cord clamping	63 (5.8)	24 (2.4)	<0.001
**Newborn**			
Birth weight, g (mean ± SD)	1239 (±351)	1156 (±368)	<0.001
<=1000 g	344 (27.8)	441 (39.3)	
> 1000 g	800 (72.2)	573 (60.7)	
Male sex	601 (52.6)	528 (52.0)	0.768
Average Apgar at 10 min (average ± SD)	9.25 (±1.39)	8.95 (±1.62)	<0.001
Oxygen therapy	798 (72.5)	754 (77.1)	0.008
Tracheal intubation	582 (50.8)	684 (66.7)	<0.001
**Anemia**			
Hb at birth, g/L (mean ± SD)	17.06 (±1.53)	13.38 (±1.50)	<0.001

Lowest Hb level during hospitalization, g/dL (mean ± SD)	10.83 (±2.60)	9.66 (±4.04)	<0.001
Hb at discharge, g/dL (mean ± SD)	11.25 (±2.19)	10.73 (±1.64)	<0.001
Transfusion	376 (31.7)	580 (55.3)	<0.001
Number of RBC transfusions (mean ± SD)	1.92 (±1.41)	2.29 (±1.78)	<0.001
No erythropoietin	623 (55.6)	478 (48.1)	<0.001
**Neonatal morbidity**			
Early neonatal morbidity **	177 (15.7)	220 (21.8)	<0.001
Neonatal morbidity	229 (21.1)	264 (29.1)	<0.001
Severe bronchopulmonary dysplasia	40 (3.4)	40 (3.9)	0.477
Stage 2-3 Necrotizing enterocolitis	3 (0.4)	8 (1.5)	0.066
Retinopathy - stage 3-4	90 (7.6)	119 (11.0)	0.003
Severe brain abnormalities	86 (6.9)	136 (12.1)	<0.001
Death (before discharge)	89 (6.9)	133 (12.1)	<0.001
**2-year outcome**			
Cerebral palsy at two-years	36 (4)	44 (6)	0.042
Pathological ASQ at two-years	452 (53.4)	383 (56.7)	0.142
Survival without neuromotor or sensory impairment	452 (53.4)	383 (56.7)	0.142

Table S4 Suppl: Sensibility, specificity, positive predictive value (PPV), negative predictive (NPV) of different Hb's threshold on outcome.

	Neonatal morbidity or death			
Hb threshold (g/dL)	Se [CI95%]	Sp [CI 95%]	PPV [CI95%]	NPV [CI95%]
10	0.028 [0.015-0.051]	0.985 [0.978-0.990]	0.314 [0.174-0.494]	0.804 [0.785-0.821]
12	0.1158 [0.0869-0.1524]	0.9507 [0.9386-0.9605]	0.368 [0.2848-0.4593]	0.8129 [0.7943-0.8301]
14	0.3753 [0.3278-0.4252]	0.7568 [0.7349-0.7775]	0.2764 [0.2394-0.3166]	0.8303 [0.8099-0.8490]
15,2	0.4458 [0.3964-0.4962]	0.4432 [0.4188-0.4679]	0.1654 [0.1439-0.1893]	0.7636 [0.7348-0.7903]
	Survival without morbidity			
	Se [CI95%]	Sp [CI95%]	PPV [CI95%]	NPV [CI95%]
10	0.0319 [0.0189-0.0524]	0.9852 [0.9775-0.9903]	0.4102 [0.2598-0.5780]	0.7596 [0.7402-0.7780]
12	0.1177 [0.0915-0.1500]	0.9517 [0.9396-0.9616]	0.4402 [0.3555-0.5285]	0.7701 [0.7505-0.7886]
14	0.3952 [0.3523-0.4396]	0.7641 [0.7420-0.7848]	0.3504 [0.3113-0.3915]	0.7969 [0.7754-0.8168]
15,2	0.4291 [0.3855-0.4738]	0.4383 [0.4135-0.4634]	0.1974 [0.1744-0.2225]	0.7045 [0.6745-0.7329]
	Outcome at two years (PC or ASQ < - 2DS			
	Se [CI95%]	Sp [CI95%]	PPV [CI95%]	NPV [CI95%]
10	0.0176 [0.0103-0.0297]	0.9875 [0.9745-0.9941]	0.6521 [0.4282-0.8281]	0.4321 [0.4066-0.4580]
12	0.0554 [0.0414-0.0735]	0.9470 [0.9260-0.9625]	0.5802 [0.4654-0.6873]	0.4315 [0.4055-0.4578]
14	0.2747 [0.2452-0.3063]	0.7585 [0.7231-0.7908]	0.6005 [0.5497-0.6492]	0.4419 [0.4124-0.4718]
15,2	0.5424 [0.5082-	0.4236 [0.3852-	0.5542 [0.5196-	0.4121 [0.3744-

	0.5763]	0.4630]	0.5883]	0.4508]

Survival at two years without sequellae (no PC and ASQ <_2DS)

	Se [CI95%]	Sp [CI95%]	PPV [CI95%]	NPV [CI95%]
10	0,023 [0,015-0,034]	0,988 [0,975-0,994]	0,758 [0,574-0,883]	0,374 [0,352-0,398]
12	0,075 [0,060-0,093]	0,947 [0,926-0,963]	0,704 [0,611-0,784]	0,378 [0,354-0,402]
14	0,309 [0,282-0,338]	0,759 [0,723-0,791]	0,684 [0,640-0,724]	0,394 [0,367-0,422]
15,2	0,491 [0,461-0,521]	0,576 [0,537-0,615]	0,662 [0,628-0,694]	0,402 [0,370-0,434]

Table S5 Suppl: Correlation early level value Hb (as a Continuous Variable) on overall cohort and in subgroup GA with neurodevelopment at two years.

**Survival without neuromotor or sensory impairment**	**Overall pop N= 2158**		**22-25+6 week GA N= 185**		**26-27+6 week GA N=447**		**28-31+6 week GA N= 1553**	
	OR IC	p	OR IC	p	OR IC	p	OR IC	p
Model 1	1,0 [0.95-1.04]	1,00	0.95[0.76-1.17]	0.65	0.95[0.76-1.17]	0.92	1 [0.95.1.05]	0.81
Model 2	0,99[0,94-1,04]	0.74	0.96 [0.78-1.2]	0.77	0.96[0.78-1.2]	0.99	0.96[0.78-1.2]	0.99
Model 3	0,99 [0,94-1,04]	0,82	0.96 [0.76-1.2]	0.77	0.96[0.76-1.2]	0.69	0.96[0.76-1.2]	0.69

Percentages and p values are weighted according to GA
Abb : aOR: Adjusted Odds Ratio; 95%; CI: 95% Confidence Interval; PC: Cerebral Palsy; ASQ: Ages Staging Questionnaire
Model 1 : Odds Ratio adjusted for GA, antenatal corticosteroid therapy, birth weight, sex, Apgar score, neonatal morbidity, socioeconomic status
Model 2 Model 1 and transfusion
Model 3 Model 2 and Erythropoietin and delayed cord clamping
